# A Comparative Analysis of Surface Characteristics of Enamel after Conventional Acid Etching and Er,Cr:YSSG Laser Irradiation

**DOI:** 10.4317/jced.59823

**Published:** 2022-09-01

**Authors:** Shahul-Hameed Faizee, R. Piradhiba, NR. Krishnaswamy, Premila-Selvi Suganthan

**Affiliations:** 1 Professor and Head of the Department, Department of Orthodontics, Sathyabama Dental College and Hospital, Chennai; 2 Reader, Department of Orthodontics, Sri Venkateshwaraa Dental College, Puducherry-605102; 3 Professor and Head of the Department, Department of Orthodontics, Ragas Dental college and Hospital, Chennai; 4 Director, Kp Tooth Care Clinic Pvt Ltd, Kp Institute of Laser Studies, Neelankarai, Chennai

## Abstract

**Background:**

To evaluate the influence of Erbium, Chromium:Yttrium-Scandium-Gallium-Garnet (Er,Cr:YSGG) laser irradiation (2W 15Hz, 2W 25Hz) on Penetration Depth, Surface Roughness, Surface Morphology and Etching Pattern of enamel and to compare it with conventional acid etching.

**Material and Methods:**

Sixty sound human premolars extracted for orthodontic reasons were used for this study. Penetration Depth and Surface Roughness between Acid Etched and Laser Etched Enamel were evaluated using Non-contact 3D- Profilometer .Surface Morphology and the Etching Pattern were evaluated using Field Emission - Scanning Electron Microscope.

**Results:**

On comparing the maximum depth and surface roughness values among all the three groups, highest was found in the acid-etched group, followed by the 2W 15Hz laser–etched group and the least was with the 2W 25Hz laser–etched group. The laser etching of enamel surface with an Er,Cr:YSGG laser system showed Type 3 etching pattern, whereas acid etched surface showed Type 1 etching pattern as described by Silverstone (1974).

**Conclusions:**

Er,Cr:YSGG laser irradiation produces favourable etching pattern and micro-irregularities that are suitable for resin penetration. Moreover, due to its decreased surface roughness and penetration depth, laser etching can significantly decrease the probability of formation of white spot lesions which is proved to be the potential disadvantage of conventional acid etching. Hence, laser etching can be preferred over the conventional phosphoric acid etching for orthodontic bonding.

** Key words:**Laser, Er,Cr:YSGG, Surface Roughness, Etching Pattern,Penetration Depth,SEM, Laboratory Research, Acid etching.

## Introduction

For many years phosphoric acid etching has been widely used for bonding orthodontic brackets. However, increased decalcification and white spot formation has been reported owing to the formation of morphologically porous layer of 5 – 50 μm depth with loss of fluoride rich enamel surface layer about 0.2 to 25 μm (1). Approximately 96% patients undergoing fixed appliance therapy show signs of enamel demineralization (2). Moreover, discoloration of enamel surface after de-bonding has been reported due to resin penetration that could significantly compromise the aesthetics of the smile (3).

Throughout the past years, many studies have focused on finding alternative methods that are less damaging to the tooth structure and simultaneously yield optimum bond strength (4-6).

Recently, attention has been driven to the practice of laser etching. Since 1964, several types of lasers have been introduced for use in dentistry; for example, ruby, neodymium-doped yttrium aluminium garnet (Nd: YAG), carbon dioxide, and erbium lasers (7).

Compared to the other lasers, the Erbium laser is the most effective for hard tissue ablation with minimal thermal effects on the pulp (8,9). It has attracted significant attention as it has the demineralisation resistance property by increasing the calcium-to-phosphorous ratio on the enamel surface (10,11). Berk 2008 (12) evaluated laser-irradiated enamel surfaces with different power outputs (0.5, 0.75, 1, 1.5, and 2W at a constant frequency of 20Hz). He concluded that 1.5W and 2W laser irradiation yielded optimum Bond strength and can be an alternative to conventional acid etching.

From the earlier studies, it has been perceived that the ideal dosimetry for laser irradiation of enamel surface for achieving optimum bond strength and demineralisation resistance are predominantly 1.5W/20Hz, 2W/20Hz (9,10,12). Accordingly in our previous study (13), Laser irradiation of enamel surface (2W 15 Hz, 2W 25 Hz) revealed significant demineralization resistance and also obtained optimum bond strength similar to that of acid etching.

However, there are not many evidences comparing the penetration depth, and surface roughness of laser conditioned surface with acid etched enamel surface. Also, there are no conclusive evidences about the effect of frequency variations. (Pulse frequency represents the number of pulses that have equal pulse energy with each other delivered to target tissue per second).

Therefore, pulse frequency–output power combinations of the Er,Cr:YSGG laser that could yield less iatrogenic damage were sought in the present study.

Thus, the aim of the present study was to evaluate the influence of Er,Cr:YSGG laser irradiation with different pulse frequency (2W 15 Hz and 2W 25 Hz) on Penetration Depth, Surface Roughness, Surface Morphology and Etching Pattern of enamel and to compare it with conventional acid etching.

## Material and Methods

**Figure 1:**
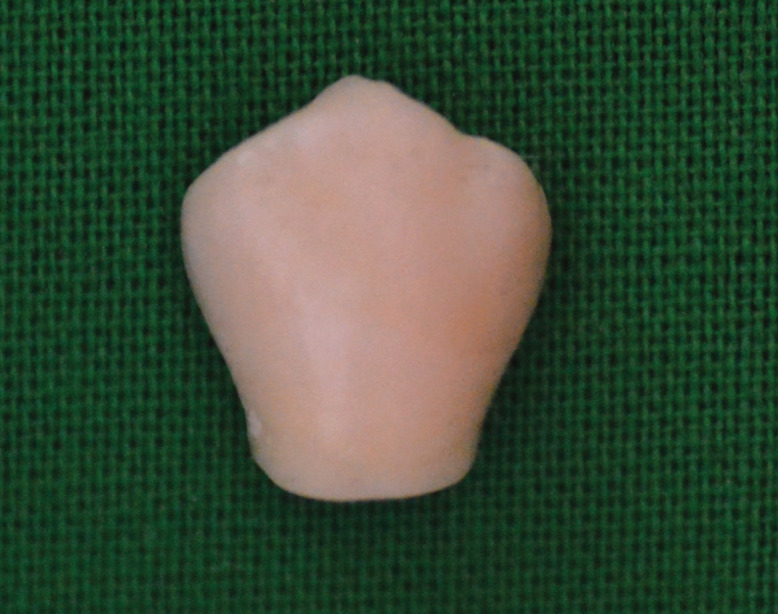
Tooth Sectioning.

-Sample description:

Sixty premolar teeth extracted for orthodontic treatment were collected and the teeth with enamel hypoplasia, caries, and cracks were excluded. The samples were then stored in distilled water. The samples were sectioned as illustrated in Figure 1.

-Group allocation:

60 teeth were allocated into three groups of 20 teeth in each as follows:

• Group I (Acid Etching)

• Group II (Laser 2W 15HZ)

• Group III (Laser 2W 25HZ) 

In Group I, the buccal enamel surface was etched with 37% phosphoric acid for 30 s and rinsed with water for 15 seconds and dried for 10 seconds.

In Group II & III, Er, Cr: YSGG laser was used for etching the enamel surface with a power output of 2W 15Hz and 2 W 25Hz respectively for 30 sec at a working distance of 5-7 mm.

-Study methodology

**Figure 2:**
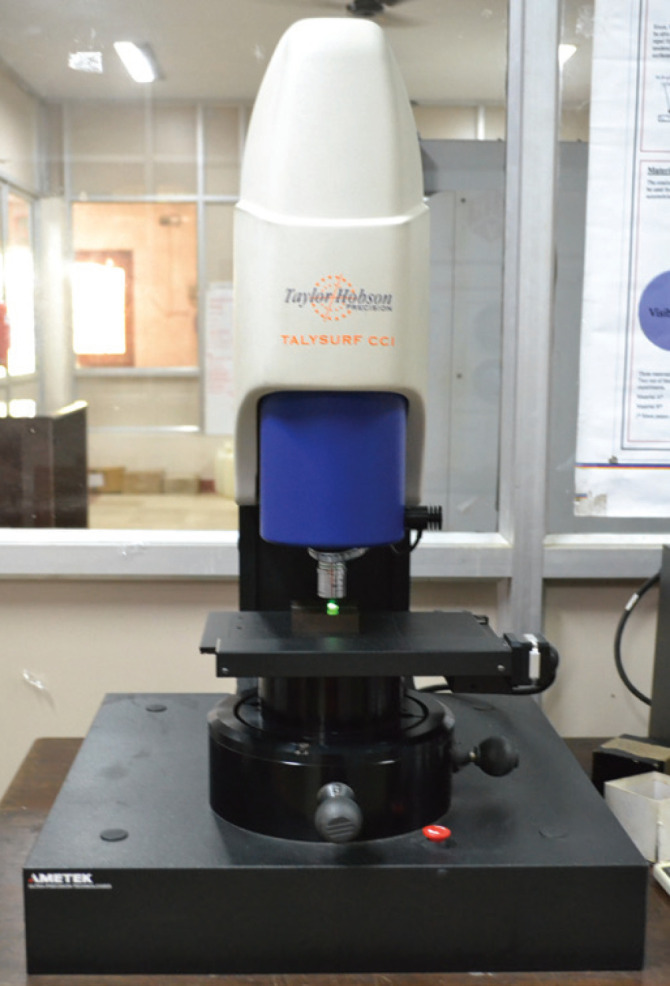
3D-Profilometer (THE TALYSURF CCI LITE).

Evaluation of Penetration Depth and Surface Roughness:

10 sectioned teeth from each group were randomly selected and analysed with 3D-PROFILOMETER (The Talysurf CCI Lite, Tayler-Hobson Co, England) (Fig. 2) to assess the penetration depth and surface roughness between acid etched and laser etched enamel surfaces.The instrument was calibrated using a standard reference specimen, and then set to travel at a speed of 0.5mm/sec for traveling length of 0.1 to 1.3 mm. The profilometer produced a tracing for calculating the surface roughness (Ra value) and maximum depth of penetration. The acquired results were subjected to statistical analysis.

**Figure 3:**
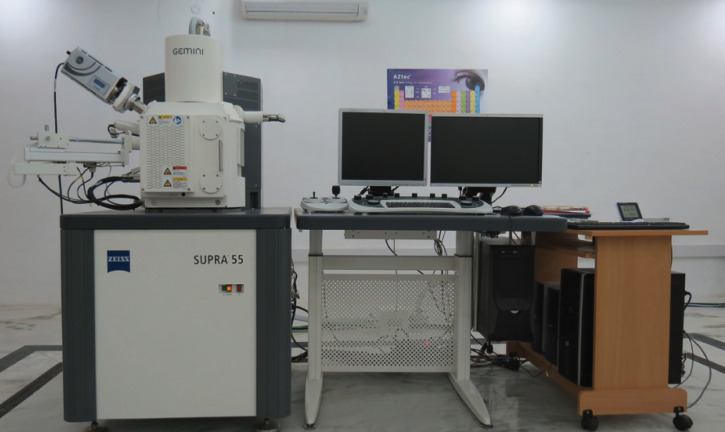
Field Emission - Scanning Electron Microscope (FE-SEM, SUPRA 55VP).

Evaluation of Surface Morphology and Etching Pattern:

10 sectioned teeth from each group were randomly selected and analyzed with Field Emission - Scanning Electron Microscope (FE-SEM, SUPRA 55VP, Carl Zeiss, Germany) (Fig. 3). The sectioned teeth were fixed onto a specimen holder, then dehydrated and sputter-coated with silver to increase thermal conduction, reduce beam penetration and enhance edge resolution.

Samples were observed under SEM. For each specimen, five micro photographs with different magnifications (x1000, x2500, x20000) were made. Using the calibrated X and Y axis markings on the microscope, the specimen was moved alternately 1 mm to the right and 1 mm to the left and the photographs repeated. A total of 15 photographs per tooth were taken.

## Results

The acquired results were subjected to statistical analysis using the SPSS Vs. 19 (IBM, USA, 2010). The Normality tests Kolmogorov Smirnov and Shapiro Wilks test results showed that all the variables followed normal distribution. To compare mean values between the three groups one way ANOVA is used; followed by Tukey’s HSD post hoc tests for pair wise comparison of mean values (If *P*-Value is <0.05 then it was considered as statistically significant).

Evaluation of penetration depth and surface roughness:

 The mean maximum depth (μm) and surface roughness (Ra) values are presented in Table 1. There were significant differences in the values between the groups (*P* < 0.001). The control group exhibited the highest depth and surface roughness values, followed by 2W15Hz and 2W 25 Hz laser groups in descending order. The acid‑etched group exhibited significantly higher values compared to the two laser groups (*P* < 0.05); however, the difference between the laser groups was not significant (Table 2).

**Table 1:**
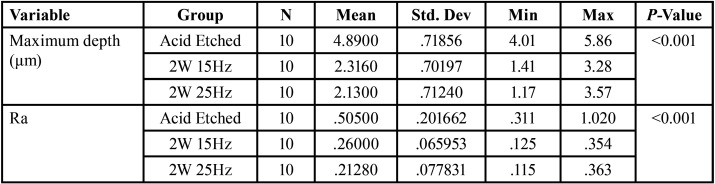
One way anova to compare men values between three groups.

**Table 2:**
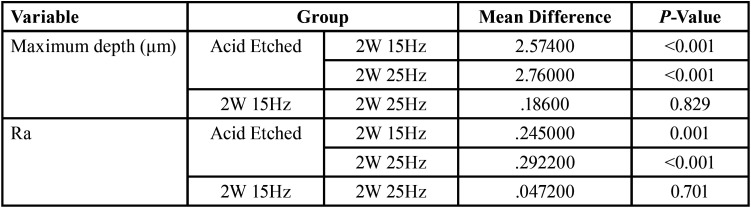
Tukey HSD post hoc tests for multiple comparisons.

Evaluation of surface morphology and etching pattern

**Figure 4:**
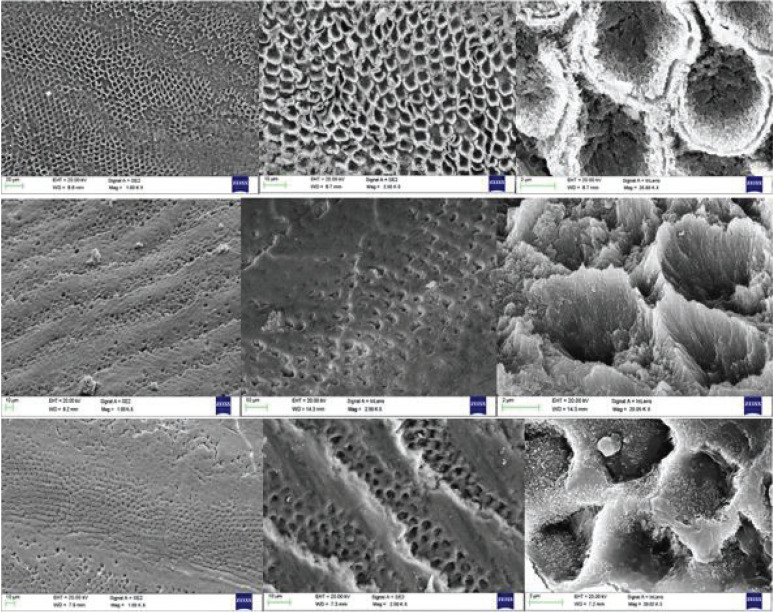
Type I Etching Pattern Shown With 37% Phosphoric Acid Etching and Type III Etching Pattern Shown With 2w 15 Hz & 2W 25 Hz Laser Etching.

Acid etched group samples showed a typical Type 1 etching pattern (Fig. 4) where prism cores were preferentially removed, leaving prism peripheries intact as described by Silverstone et at14,15. Whereas, the laser etched samples (2W 15 Hz &25Hz) showed Type 3 etching pattern (Fig. 4) where there was a more random pattern, areas of which corresponded to types 1 and 2 damage together with regions in which the pattern of etching could not be related to prism morphology.

## Discussion

Lasers have become an integral part of Orthodontists’ armamentarium. They have proved to be useful as adjunct to conventional treatment modalities in various soft and hard tissue intraoral procedures with better patient acceptance. Laser etching has been advocated as an alternative to acid etching to counteract the iatrogenic effects such as enamel demineralisation, discolouration (16).

Er,Cr:YSGG, has a high absorption coefficient in water and enamel because laser wavelengths operate in the region of the major absorption peak for water (2790 nm), and are thus the most suited to hard tissue ablation treatments (17). This led researchers to explore its use in enamel etching.

From the previous studies, it can be inferred that laser irradiation with power output of 1.5- and 2-W yielded Optimum Bond strength and adequate demineralisation resistance (11,12,18). But, there are not much evidences comparing the surface roughness, depth of penetration, surface morphology and etching pattern between laser etching and conventional acid etching. Hence, we performed a comparative analysis on the enamel surface characteristics after Er,Cr:YSGG laser irradiation and conventional acid etching. Additionally, we also evaluated the effect of varying the frequency of laser (2W 25Hz, 2W 15Hz) on the surface characteristics of enamel.

Penetration depth and surface roughness between acid etched and laser etched enamel was evaluated using 3D- Profilometer. It is a type of measurement interferometer which uses an innovative, patented correlation algorithm to find the coherence peak and phase position of an interference pattern produced by the precision optical scanning unit. The highest mean surface roughness (Ra) was found in the acid-etched group (0.51), followed by the 2W 25Hz (0.21) and the least was with the 2W 15Hz laser etched group (0.26).

The reason for comparatively less penetration depth in 2W25Hz laser etched group than 2W15Hz laser etched group may be attributed to the fact that if the frequency is increased, there is a decrease in the amount of energy carried by each pulse, thereby increasing the surface area with less penetration depth and thus, decreased surface roughness. These results are in accordance with Visuri *et al*. (8) who concluded that the laser etching of enamel surface revealed decreased penetration depth compared to acid etched surface.

The surface morphology and etching pattern between acid etched and laser etched enamel was evaluated using Field Emission Scanning Electron Microscope (FESEM). FESEM allows surface examination down to nanometre scales in high vacuum. It consists of an Energy Dispersive x-ray Spectrometer (EDS), an Electron Back Scattered Diffraction (EBSD) system and Nanometre Pattern Generation System (NPGS) for crystal orientation, phase mapping, elemental analysis and mapping respectively.

The effect was studied under the magnifications of x1000, x2500, x20000. Acid etched enamel surface (Fig. 4) showed a typical Type 1 etching pattern where prism cores were preferentially removed, leaving prism peripheries intact as described by Silverstone (14). This was in accordance with the previous studies by Johnston (19) and Retief *et al*. (20) who found that etching for 30 seconds produced Type I or an ideal etch pattern, that is, preferential dissolution of enamel prism cores and boundaries.

Whereas, laser etched enamel surfaces (2W 15HZ, 2W 25 HZ ) (Fig. 4) showed type 3 etching pattern which corresponds to the combination of types 1 and 2 where the pattern of etching could not be related to prism morphology resulting in a uniform roughened surface. This finding was supported by the Patrícia *et al*. (21).

The random pattern in laser etched surfaces may be due to selective removal of enamel hydroxyapatite crystals resulting in uniform rough surface that would enhance the micromechanical retention (7,9). Furthermore, parameter factors Varying pulse width, pulse mode and spot size can produce significant changes in enamel and dentin surface morphology such and wavelength specificity relate to the degree of change that can be induced to enamel (22).

From the results of the study, it can be inferred that laser etching preserves the integrity of enamel by decreasing the penetration depth and the surface roughness. Additionally, it produces favourable etching pattern. Owing to these results, iatrogenic effects of acid etching such as discoloration of enamel surface, White Spot lesions can be negated by laser etching.

Further studies using a larger sample size with different frequency / power combinations are warranted to evaluate the precise efficacy of laser systems in enamel irradiation. In addition, long term *in vivo* studies comparing conventional acid etching and laser etching should be conducted to know its effect on a clinical setting.

## Conclusions

• Laser ablation produces favourable etching pattern and less iatrogenic damage by decreasing the penetration depth and the surface roughness.

• 2W 25Hz can be preferred over 2W 15Hz because 2W 25Hz laser etched surface has decreased penetration depth and surface roughness over 2W 15Hz.

•Hence, Er,Cr:YSGG laser irradiation of enamel surface can be preferred over the conventional phosphoric acid etching in orthodontic preparation.

-Clinical significance.

Lasers have become a significant modality of treatment for many clinical conditions that dentists treat on a daily basis. In Orthodontics, Laser conditioning of enamel can overcome the iatrogenic effects of conventional acid etching such as White spot lesions, discolouration due to its decreased penetration depth, surface roughness.
